# Mortalidade Pós-Internação na Insuficiência Cardíaca: Lições de uma Análise por Múltiplas Causas

**DOI:** 10.36660/abc.20250444

**Published:** 2025-09-11

**Authors:** Danielle Louvet Guazzelli, Monica S. Avila

**Affiliations:** 1 Universidade de São Paulo Faculdade de Medicina Hospital das Clínicas Instituto do Coração São Paulo SP Brasil Universidade de São Paulo Faculdade de Medicina Hospital das Clínicas Instituto do Coração, São Paulo, SP – Brasil

**Keywords:** Insuficiência Cardíaca, Mortalidade, Causas de Morte

A insuficiência cardíaca (IC) está entre as principais causas de hospitalização e morte, mas as causas diretas que determinaram óbito, sobretudo após uma internação por descompensação, se mostram hoje muito mais heterogêneas do que se supõe. O artigo "Análise da Mortalidade por Múltiplas Causas na Insuficiência Cardíaca de Acordo com a Fração de Ejeção"^1^ publicado nesta edição dos Arquivos Brasileiros de Cardiologia, realizou uma avaliação retrospectiva de uma coorte prospectiva em 519 pacientes internados por IC descompensada entre 2011 e 2019 e que tiveram as declarações de óbito analisadas pelo método de múltiplas causas (MCOD). Em uma média de 2,9 anos de seguimento, 52 % vieram a óbito. Contudo, apenas 36 % das 977 menções registradas nos atestados eram de natureza cardiovascular, proporção nitidamente inferior aos 50% descritos previamente na literatura nacional.^2^ Sepse, a própria IC e pneumonia despontaram como causas específicas mais frequentes e, diferentemente de estudos que atribuem pior prognóstico à fração de ejeção reduzida, a sobrevida não diferiu entre os grupos com fração de ejeção preservada, levemente reduzida ou reduzida. Vale notar ainda que o código I50 (IC), habitual líder nas certidões brasileiras (≈ 23 % das menções em estudos prévios).^3^ ocupou apenas a terceira posição, superado por septicemia e pneumonia.

O período pós-alta se caracteriza por vulnerabilidade acrescida e múltiplas ameaças concorrentes. Registros internacionais já apontam mortalidade de 20–30 % no primeiro ano, com predomínio crescente de causas não cardiovasculares nos óbitos tardios.^4,5^ O estudo brasileiro reforça essa transição: embora a letalidade hospitalar tenha sido de 14,5 %, infecções e complicações respiratórias responderam pela maior parte das mortes após a alta, padrão igualmente observado em ensaios com inibidores de SGLT2 e sacubitril/valsartana.^6,7^

O artigo delineia também perfis de risco distintos conforme o fenótipo da fração de ejeção. Pela análise de correspondência, neoplasias se associaram à IC com fração preservada, distúrbios endócrino-metabólicos à forma levemente reduzida e doença pulmonar crônica à forma reduzida — distribuição de comorbidades ainda escassamente descrita na literatura.^8^ Além disso, os autores assinalam ser este o primeiro trabalho brasileiro (e um dos poucos internacionais) a cruzar o MCOD com categorias de fração de ejeção, sublinhando a pertinência de estender esse modelo analítico a coortes maiores e mais diversas.

Do ponto de vista metodológico, o uso do MCOD permitiu superar a limitação de analisar apenas a causa básica, revelando a rede causal completa que culmina no óbito. A integração de eventos intra-hospitalares e pós-alta oferece uma visão longitudinal rara em estudos que não contam com registros populacionais extensos. Entre as limitações, se destaca o caráter unicêntrico (hospital privado de alta complexidade), a ausência de 7 % dos atestados de óbito e o potencial de erro na codificação de texto livre; ainda assim, a convergência com relatórios epidemiológicos nacionais apoia a validade externa dos achados.

Do ponto de vista assistencial, o trabalho reforça que o seguimento da IC deve ser intrinsecamente multidisciplinar. Cardiologistas precisam atuar em conjunto com infectologistas, pneumologistas, oncologistas e nutricionistas para prevenir, rastrear e tratar comorbidades que impactam substancialmente a sobrevida.^9^ Programas de transição que combinam visita precoce, telemonitoramento e imunização são importantes para redução de reinternações.^10^

Em síntese, a análise demonstra que a mortalidade na IC é modulada por um conjunto de condições infecciosas, respiratórias, oncológicas e metabólicas que acompanham o paciente ao longo de toda a continuidade de cuidados. A identificação sistemática e o manejo proativo dessas comorbidades, paralelamente ao tratamento cardiológico baseado em evidências, devem ser considerados parte integrante da prática clínica atual.
