# Supplementary material for: Mortalidade Pós-Internação na Insuficiência Cardíaca: Lições de uma Análise por Múltiplas Causas
Source: Arq Bras Cardiol. 2025 Sep 11;122(9):e20250444. [Article in Portuguese] doi: 10.36660/abc.20250444 (PMC12671601; doi:10.36660/abc.20250444)
Supplement: Translation

# Translation: Post-discharge Mortality in Heart Failure: Insights from a Multiple-Cause Analysis

**DOI:** 10.36660/abc.20250444i

**Authors:** Danielle Louvet Guazzelli, Monica S. Avila

**Affiliations:** 1 São Paulo SP Brazil Universidade de São Paulo Faculdade de Medicina Hospital das Clínicas Instituto do Coração, São Paulo, SP – Brazil

**Keywords:** Heart Failure, Mortality, Cause of Death

Heart failure (HF) ranks among the leading causes of hospital admission and death. Nevertheless, the factors precipitating mortality—particularly after a decompensation-related admission—are far more heterogeneous than once assumed. In the article "Multiple Cause of Death Analysis in Heart Failure According to Ejection Fraction"^1^ published in this issue of the *Arquivos Brasileiros de Cardiologia*, 519 patients hospitalised for decompensated HF between 2011 and 2019 had their death certificates examined with a multiple-cause-of-death (MCOD) approach. Over a mean follow-up of 2.9 years, 52 % of patients died; however, only 36 % of the 977 recorded mentions were cardiovascular, a proportion clearly lower than the 50% previously reported in Brazilian series.^2^ Sepsis, HF itself, and pneumonia were the most frequent specific causes, and—contrary to studies linking reduced ejection fraction to worse prognosis—survival did not differ across preserved, mildly reduced, or reduced ejection-fraction groups. Remarkably, code I50 (HF), historically the leading line on Brazilian death certificates (≈ 23 % of mentions),³ ranked only third, surpassed by sepsis and pneumonia.

The post-discharge period is characterized by heightened vulnerability and multiple competing threats. International registries already show 20–30 % mortality in the first year, with a growing dominance of non-cardiovascular causes in late deaths.^4,5^ The Brazilian study reinforces this shift. Although in-hospital lethality was 14.5 %, infections and respiratory complications accounted for most post-discharge deaths, mirroring patterns seen in trials of SGLT2 inhibitors and sacubitril/valsartan.^6,7^

The article also delineates distinct risk profiles according to ejection-fraction phenotype. Correspondence analysis linked neoplasms to HF with preserved ejection fraction, endocrine–metabolic disorders to mildly reduced ejection fraction, and chronic lung disease to reduced ejection fraction—a distribution of comorbidities still sparsely documented in the literature.^8^ Moreover, the authors note that this is the first Brazilian work (and one of the few worldwide) to cross MCOD data with ejection-fraction categories, underlining the need to extend this analytic model to larger and more diverse cohorts.

Methodologically, the MCOD approach transcended the limitation of analyzing only the underlying cause, revealing the entire causal chain culminating in death. Integrating in-hospital and post-discharge events affords a longitudinal view rarely attainable in studies lacking extensive population databases. Limitations include the single-center design (a high-complexity private hospital), absence of 7 % of death certificates, and potential miscoding of free text; nonetheless, convergence with national epidemiological reports supports the external validity of the findings.

From a clinical perspective, the work underscores that HF follow-up must be intrinsically multidisciplinary. Cardiologists need to collaborate with infectious-disease, pulmonary, oncology, and nutrition specialists to prevent, screen, and treat comorbidities that substantially influence survival.^9^ Transitional-care programs combining early follow-up, remote monitoring, and immunization are important to reduce readmissions.^10^

In summary, the analysis demonstrates that mortality in HF is driven by a cluster of infectious, respiratory, oncological, and metabolic conditions that accompany the patient throughout the continuum of care. Systematic identification and proactive management of these comorbidities—alongside evidence-based cardiac therapy—should be regarded as integral components of contemporary clinical practice.
